# A Novel Root-Knot Nematode Resistance QTL on Chromosome Vu01 in Cowpea

**DOI:** 10.1534/g3.118.200881

**Published:** 2019-02-28

**Authors:** Arsenio D. Ndeve, Jansen R. P. Santos, William C. Matthews, Bao L. Huynh, Yi-Ning Guo, Sassoum Lo, Maria Muñoz-Amatriaín, Philip A. Roberts

**Affiliations:** * Deptartment of Nematology; † Department of Botany and Plant Sciences, University of California, Riverside, CA 92521,; § Departamento de Fitopatologia, Universidade de Brasilia, Brasilia, DF, 70910-900 Brazil

**Keywords:** Meloidogyne spp., Quantitative Trait Loci, Vigna unguiculata

## Abstract

The root-knot nematode (RKN) species *Meloidogyne incognita* and *M. javanica* cause substantial root system damage and suppress yield of susceptible cowpea cultivars. The narrow-based genetic resistance conferred by the *Rk* gene, present in some commercial cultivars, is not effective against *Rk*-virulent populations found in several cowpea production areas. The dynamics of virulence within RKN populations require a broadening of the genetic base of resistance in elite cowpea cultivars. As part of this goal, F${}_{1}$ and F${}_{2}$ populations from the cross CB46-Null (susceptible) x FN-2-9-04 (resistant) were phenotyped for *M. javanica* induced root-galling (RG) and egg-mass production (EM) in controlled growth chamber and greenhouse infection assays. In addition, F${}_{2:3}$ families of the same cross were phenotyped for RG on field sites infested with *Rk*-avirulent *M. incognita* and *M. javanica*. The response of F${}_{1}$ to RG and EM indicated that resistance to RKN in FN-2-9-04 is partially dominant, as supported by the degree of dominance in the F${}_{2}$ and F${}_{2:3}$ populations. Two QTL associated with both RG and EM resistance were detected on chromosomes Vu01 and Vu04. The QTL on Vu01 was most effective against aggressive *M. javanica*, whereas both QTL were effective against avirulent *M. incognita*. Allelism tests with CB46 x FN-2-9-04 progeny indicated that these parents share the same RKN resistance locus on Vu04, but the strong, broad-based resistance in FN-2-9-04 is conferred by the additive effect of the novel resistance QTL on Vu01. This novel resistance in FN-2-9-04 is an important resource for broadening RKN resistance in elite cowpea cultivars.

Root-knot nematode (RKN) species, particularly *Meloidogyne incognita* and *M. javanica*, cause substantial damage to root systems and suppress yield of susceptible cowpea (Vigna unguiculata L. Walp) cultivars by impairing water and nutrient uptake, and the partitioning and translocation of photo-assimilates (Bird and Loveys 1975; McClure 1977; Taylor and Sasser 1978; Williamson and Hussey 1996; Sikora *et al.* 2005). Host-plant resistance is an important strategy to mitigate the impact of nematode infestation (Hall and Frate 1996; Roberts 1992; Ehlers *et al.* 2000b; Castagnone-Sereno 2002; National Research Council 2006), both in Africa where access to agronomic inputs including nematicides is limited (Sasser 1980; Luc *et al.* 2005), and in developed agriculture where resistant varieties are the best option economically (Ehlers *et al.* 2000b).

Narrow-based resistance conferred by a single dominant gene *Rk* has provided protection against RKN in cowpea agricultural systems worldwide (Amosu and Franckowiak 1974; Singh and Reddy 1986; Helms *et al.* 1991; Fery *et al.* 1994; Roberts *et al.* 1995; Roberts *et al.* 1996; Roberts *et al.* 1997; Ehlers and Hall 1997; Ehlers *et al.* 2009). The resistance conferred by gene *Rk* is highly effective against avirulent forms of RKN populations (Roberts *et al.* 1995; Hall and Frate 1996; Roberts *et al.* 1997; Ehlers *et al.* 2000a,b; Roberts *et al.* 2013), but *Rk*-virulent and aggressive forms of common RKN species have been identified (Swanson and Van Gundy 1984; Roberts *et al.* 1995; Hall and Frate 1996; Roberts *et al.* 1997; Petrillo *et al.* 2006). Selection for virulence to *Rk* (Roberts *et al.* 1997; Petrillo and Roberts 2005; Petrillo *et al.* 2006) has prompted efforts to broaden the genetic base of resistance in elite cowpea cultivars (Hall and Frate 1996; Roberts *et al.* 1996; Roberts *et al.* 1997; Ehlers *et al.* 2000b; Roberts *et al.* 2013). The threat imposed by virulence in RKN populations led to the discovery of new resistance genes, *Rk${}^{2}$* - with a dominant effect (Roberts *et al.* 1996; Roberts *et al.* 1997; Ehlers *et al.* 2000b) and *rk${}^{3}$* - with recessive and additive effect (Roberts *et al.* 1996; Ehlers *et al.* 2000a), to broaden the genetic base of resistance, and advanced breeding materials with a combination of these resistance genes have shown promising performance under RKN infestation (Roberts *et al.* 1996; Roberts *et al.* 1997; Ehlers *et al.* 2000b; Ehlers *et al.* 2002). For example, the additive effect of gene *Rk${}^{2}$* in breeding line IT84S-2049 (which also carries gene *Rk*) contributes substantially to an enhanced resistance to *Rk*-virulent populations of *M. incognita* and to *M. javanica* compared to gene *Rk* alone (Roberts *et al.* 1996; Roberts *et al.* 1997; Roberts *et al.* 2005). The *rk${}^{3}$* locus modifies slightly the resistance provided by gene *Rk* under *Rk*-virulent RKN isolates (Ehlers *et al.* 2000b), and it is present in cowpea cv. CB27 (Ehlers *et al.* 2000a). Although the contribution of these resistance genes is known, their individual action is not clearly understood. However, these examples of resistance gene combinations have shown that broad-based genetic resistance can be developed through effective gene pyramiding of independent sets of resistance genes from distinct genetic sources (Ehlers *et al.* 2002).

The *Rk* locus has been mapped on chromosome Vu04 (Huynh *et al.* 2016), the previous cowpea linkage group 11 of the cowpea consensus genetic map (Lucas *et al.* 2011; Muñoz-Amatriaín *et al.* 2017). This genomic region and flanking markers associated with RKN resistance within this region are important resources for introgressing this resistance into elite cowpea cultivars. Also, markers flanking the resistance in this genomic region can be utilized as a reference to decipher the genetic relationship between the resistance conferred by gene *Rk* and potential novel sources of resistance to RKN. A broad-based resistance to RKN has been identified through a series of field, greenhouse and seedling growth pouch tests in a cowpea accession FN-2-9-04 from Mozambique (Ndeve *et al.* 2018). This accession carries higher levels of resistance to avirulent *M. incognita* and *M. javanica* than that conferred by the *Rk* gene alone. The performance of FN-2-9-04 under *M. javanica* infestation was contrasted to cowpea breeding lines and cowpea cultivars carrying sets of RKN resistance genes, including *RkRk/$Rk^{2}Rk^{2}$*, *RkRk/$rk^{3}rk^{3}$*, *RkRk/$Rk^{2}Rk^{2}$*/gg and IT84S-2049 which indicated that the RKN resistance in accession FN-2-9-04 is unique. Therefore, to characterize the resistance in FN-2-9-04, genetic analyses were conducted to determine its genomic architecture and localization through genetic linkage analysis and QTL mapping.

## Materials and Methods

### Plant materials

Four F${}_{1}$, three F${}_{2}$ and one F${}_{2:3}$ populations (Table 1) were developed under greenhouse conditions at the University of California Riverside (UCR). Accession FN-2-9-04 was crossed with CB46-Null, CB46, Ecute and INIA-41. A single F${}_{1}$ seed from each of the crosses CB46-Null x FN-2-9-04, CB46 x FN-2-9-04 and INIA-41 x FN-2-9-04 was grown to derive three independent F${}_{2}$ populations, and 150 F${}_{2}$ lines of population CB46-Null x FN-2-9-04 were advanced to generate 150 F${}_{2:3}$ families (Table 1). Four F${}_{1}$ populations (CB46-Null x FN-2-9-04, CB46 x FN-2-9-04, INIA-41 x FN-2-9-04, Ecute x FN-2-9-04) and subsets of their F${}_{2}$ populations were phenotyped for root-galling and egg-mass production in greenhouse and seedling growth-pouch screens, respectively, following infection with nematode isolates listed in Table 1. Five to ten seeds per F${}_{1}$ population were also screened in each test. The subsets of F${}_{2}$ populations and F${}_{2:3}$ families (Table 1) also were phenotyped for root-galling in field experiments.

**Table 1:** Cowpea populations used for inheritance studies and QTL mapping, their size, phenotyping conditions, target trait, nematode isolate used and year of testing

Exp *^a^*	Population	Size	Environment	Trait	Nematode isolate	Year
1	*^b^*CB46-Null/FN-2-9-04 (F${}_{2}$)	163	SGP-UCR	EM	*M.j*	2015
2	*^b^*CB46/FN-2-9-04 (F${}_{2}$)	172	SGP-UCR	EM	*M.j*	2015
3	*^b^*INIA-41/FN-2-9-04 (F${}_{2}$)	126	GH-UCR	RG	*M.j*	2015
4	*^b^*CB46-Null/FN-2-9-04 (F${}_{2}$)	177	GH-UCR	RG	*M.j*	2015
5	*^b^*CB46/FN-2-9-04 (F${}_{2}$)	197	GH-UCR	RG	*M.j*	2015
6	CB46/ FN-2-9-04 (F${}_{2}$)	400	CVARS	RG	Avr-*M.i*	2015
7	CB46/FN-2-9-04 (F${}_{2}$)	162	KARE	RG	Avr-*M.i*	2015
8	CB46-Null/FN-2-9-04 (F${}_{2:3}$)	150	SCREC	RG	*M.j*	2016
9	CB46-Null/FN-2-9-04 (F${}_{2:3}$)	150	SCREC	RG	Avr-*M.i*	2016

a Exp. = experiment; SGP = seedling growth-pouches; GH = greenhouse; RG = root-galling; EM = egg masses; Avr-*M.i* = avirulent *M. incognita* and *M.j* – *M. javanica* Project 811; UCR = University of California Riverside; CVARS = University of California Coachella Valley Agricultural Research Station; KARE = University of California Kearney Agricultural Research and Extension Center; *^b^*Experiment included the F${}_{1}$ plus Ecute x FN-2-9-04 F${}_{1}$ plants.

CB46 is a California blackeye cultivar carrying gene *Rk* (Helms *et al.* 1991), and the CB46-Null genotype is a near-isogenic breeding line (NIL) derived from CB46. This breeding line has the CB46 background, but it is susceptible (minus *Rk* via backcrossing) (Huynh *et al.* 2016). Ecute and INIA-41 are landraces and FN-2-9-04 is an accession from Mozambique. FN-2-9-04 is resistant to both the avirulent *M. incognita* isolates and *M. javanica* isolate used in this study, whereas CB46-Null, CB46, Ecute and INIA-41 are all susceptible to *M. javanica*. In addition, CB46-Null and Ecute are susceptible to the avirulent *M. incognita* isolates (Beltran and Project 77), whereas INIA-41 is resistant.

### Root-knot nematode isolates

Four RKN isolates were used to phenotype plant materials for response to infection. Three *M. incognita* isolates, Beltran, Project 77 and an equivalent isolate indigenous to CVARS are avirulent to the *Rk* gene, with little or no galling and EM production on root systems of plants carrying gene *Rk* (Roberts *et al.* 1995; Roberts *et al.* 1996; Roberts *et al.* 1997), whereas *M. javanica* isolate Project 811 is an aggressive isolate due to its enhanced parasitic ability (Ehlers *et al.* 2000b; Ehlers *et al.* 2009), inducing galling and reproducing successfully on roots of plants carrying *Rk* (Thomason and McKinney 1960; Roberts *et al.* 1997; Ehlers *et al.* 2009).

### Resistance phenotyping: egg-mass production

The F${}_{1}$ and F${}_{2}$ populations (Table 1) plus parental genotypes were phenotyped for *M. javanica* EM production in seedling growth-pouches according to Ehlers *et al.* (2000b) and Atamian *et al.* (2012). Briefly, a single seed of each F${}_{1}$ and F${}_{2}$ was planted per plastic pouch, and the plants were grown in a controlled environment chamber with day/night temperatures set at 28/22 *^o^*C under 16 h day-length. Plants were inoculated two weeks after germination with 1500 freshly hatched second-stage juveniles (J${}_{2}$) of *M. javanica*. Two days after inoculation, plants were supplied daily with fertilizer for 3-5 days using half-strength Hoagland’s solution (Hoagland and Arnon 1950). Thirty-five days after inoculation, the pouches were irrigated with erioglaucine dye (Sigma Chemical Co., St. Louis, MO, USA) to stain egg-masses, which were counted under 10X magnification.

### Resistance phenotyping: root-galling

Phenotyping for resistance to root-galling was conducted under greenhouse and field conditions in 2015 and 2016 (Table 1). In the greenhouse, the F${}_{1}$ and F${}_{2}$ populations and parental genotypes phenotyped for response to *M. javanica* egg-mass production in seedling growth-pouches (in growth chamber conditions) were then transplanted into 4L pots containing UC mix 3 soil and maintained at 28/22 *^o^*C day/night temperatures. After 21 days, each plant was inoculated with 10 ml of *M. javanica* egg suspension in water adjusted to 1000 eggs/ml. All greenhouse-grown plants were irrigated twice per day by drip-irrigation for about 90 days to allow seed production, and F${}_{2:3}$ seeds were collected from each F${}_{2}$ plant. After seed collection, the plant tops were cut at 2 – 3 cm above the soil line, and the roots were washed and scored for root-galling response under 10X magnification, using a 0 - 9 gall index (GI) modified from Bridge and Page (1980): 0 = no galls on root system; 1 = very few, small galls and hard to see; 5 = generally large galls can be seen on the root system and the taproot slightly galled, with galls of different sizes; 9 = large galls on the root system, and most lateral roots lost.

Field experiments were conducted in 2015 and 2016 at three sites (Table 1). At CVARS and KARE, 400 and 162 CB46 x FN-2-9-04 F${}_{2}$ lines, respectively, were phenotyped for root-galling response to avirulent M. incognita (isolate Project 77 at KARE and an equivalent to it at CVARS). In 2016 at SCREC parental genotypes, F${}_{2}$ and F${}_{2:3}$ populations were phenotyped for root-galling response in separate fields infested with avirulent *M. incognita* isolate Beltran or *M. javanica* (Table 3). In both experiments (Exps. 8 and 9), F${}_{2:3}$ families with 25 – 30 plants/family were planted in single plots. The *M. javanica* isolate used in the pot and seedling growth-pouch screens was the same isolate used to infest field sites. For both F${}_{2}$ and F${}_{2:3}$ generations, 25 - 30 seeds were planted on a 1.5 m-long single row plot, and 60 days after plant emergence plant tops were cut at 2 – 3 cm above the soil line, and the root systems dug and evaluated for root-galling using the same root-galling index described for the pot tests (Bridge and Page 1980).

### Inheritance of resistance and allelism test

Segregation for the FN-2-9-04 resistance to root-galling and reproduction by *M. javanica* and root-galling by avirulent *M. incognita* isolates was determined using both phenotypic (root-galling and egg-masses) and genotypic data. In addition, phenotypic data of F${}_{1}$, F${}_{2}$ and F${}_{2:3}$ populations, and SNP marker genotypes of F${}_{2}$ populations at mapped QTL regions were processed for goodness-of-fit analysis to determine the genetic model underlying resistance to RKN in FN-2-9-04. Analysis of goodness-of-fit of segregation ratio between resistant-susceptible lines in the F${}_{2}$ was performed through marker-trait association analysis using marker genotypes within mapped QTL regions (see Table 2) and phenotypic response of F${}_{2}$ and F${}_{2:3}$ populations. Each F${}_{2}$ line was scored for presence of parental alleles at each locus within the mapped QTL, and scores 2, 1 and 0 were assigned to homozygous favorable allele (BB = resistant parent), heterozygous (AB) and homozygous non-favorable allele (AA = susceptible parent), respectively. The genotype of each F${}_{2}$ line, within the QTL region, was determined as the mean score across all marker loci, and it was associated with its RG or EM phenotypic response determined at the F${}_{2}$ and F${}_{2:3}$ generations. The data for frequency distribution of genotypes (BB, AB and AA) (Table 3) were processed for goodness-of-fit analysis, and the chi-square values were determined following Yates correction for continuity (Little and Hills 1978). The numbers of genetic determinants associated with resistance were estimated using the Castle (1921) estimator of gene number, $n=\frac{{\left(P_{1}-P_{2}\right)}^{2}}{8Vg}$, where *n* is the estimated number of genes influencing the trait, $P_{1}$ and $P_{2}$ are the mean phenotypic values of the parents of the population and Vg is the genetic variance of the trait. To estimate the number of genes governing response to root-galling and egg-mass production, the Vg influencing these traits was derived as the genetic variance in the mapped QTL regions, flanked by known SNP markers.

**Table 2:** Chromosome locations of root-knot nematode (RKN) resistance determinants in cowpea accession FN-2-9-04, mapped using F${}_{2}$ and F${}_{2:3}$ populations of the cross CB46-Null x FN-2-9-04 and the F${}_{2}$ population of the cross CB46 x FN-2-9-04

Pop *^a^*	Trait	RKN	Vu	Position	Flanking markers	-log*p*	PVE (%)	A	D/A
			1	34.4	2..04038-2..26991	5.4	33.0	−1.3	0.5
F${}_{2:3}$	RG	Avr-*M.i*	4	24.7-27.6	2..44685-2..10583	20	73.3	−2.0	0.5
F${}_{2:3}$	RG	*M.j*	1	27.7-42.0	2..47796-1.0027	20	95.1	−2.3	0.3
F${}_{2}$	RG	*M.j*	1	30.3-38.7	2..32677-2..19840	20	47.3	−2.8	0.4
${\text{F}}_{2}^{b}$	RG	*M.j*	1	19.2-72.9	2..53036-2..18359	20	65.9	2.7	0.8
F${}_{2}$	EM	*M.j*	1	31.5-36.9	2..21671-2..07103	10.9	34.1	−17.0	0.5
${\text{F}}_{2}^{b}$	EM	*M.j*	1	47.1-52.1	2..21671-2..12209	8.8	24.7	−16.4	0.4

a Pop = mapping population; the F${}_{2:3}$ were phenotyped in the field whereas the F${}_{2}$ were phenotyped in greenhouse and growth chamber (seedling-growth pouches) screens; RG = root-galling; EM = egg-masses per root system; Avr-*M.i* = avirulent *M. incognita* isolate Beltran; *M.j* = *M. javanica*;

b mapping population CB46 x FN-2-9-04 phenotyped for RG and EM; Vu = cowpea chromosome pseudomolecule numbering (Lonardi *et al.* 2017); -log*p* = level of significance of the detected QTL (*P* < 0.05); PVE = percent of total phenotypic variation explained; A = additive effect of favorable alleles from the resistant parent (negative values indicate the extent of average reduction in RG or EM production due to the presence of favorable alleles); D = dominance effect due to substitution of favorable allele; and D/A = degree of dominance.

**Table 3:** Best fit segregation ratios (resistant:susceptible) in 119 and 141 F${}_{2}$ plants from crosses CB46-Null x FN-2-9-04 and CB46 x FN-2-9-04, respectively, determined using SNP marker loci at the two nematode resistance QTL regions

	Genotypes (Observed)*^a^*							
F${}_{2}$ Population	BB + AB	AA	Exp	$X^{2}$	*P*-value	Trait	Vu	Isolate
	96	23	13:3*^b^*	0.002	0.95-0.99	RG	1	
CB46-NullxFN-2-9-04	93	26	13:3*^b^*	0.56	0.25-0.50	RG	4	Avr-M.i
CB46-NullxFN-2-9-04	97	22	13:3*^b^*	0.002	0.95-0.99	RG	1	M.j
CB46-NullxFN-2-9-04	98	21	13:3*^b^*	0.04	0.75-0.90	EM	1	M.j
CB46xFN-2-9-04	111	30	13:3*^b^*	0.44	0.50-0.75	RG	1	Mj
CB46xFN-2-9-04	109	32	13:3*^b^*	1.19	0.25-0.50	EM	1	Mj

a BB = alleles from resistant parent, AB = heterozygous, AA = alleles from susceptible parent; Exp. = expected ratio; RG = root galling, EM = egg masses per root system; Vu = cowpea chromosome naming (Lonardi *et al.* 2017); Isolate = Nematode isolate; Avr = avirulent *M. incognita* Beltran, *M.j* = *M. javanica*; *^b^*also fit a 3:1 ratio.

Broad-sense heritability ($H^{2}=\frac{Vg}{Vp}$) of resistance was estimated using two methods, midparent-offspring regression analysis (Fernandez and Miller 1985; Falconer and Mackay 1996) and the phenotypic variation among F${}_{2}$ lines and among F${}_{2:3}$ families accounted for by Vg∗ at the QTL regions associated with resistance. The proportion of phenotypic variance, Vp∗, in root-galling or egg-masses attributed to genetic factors, Vg∗, was estimated using SNP marker genotype scores (Vgs) and SNP marker effects (SNPeff) at the mapped QTL regions plus the observed root-galling or egg-masses phenotypes using the algorithm: $Vp*=\frac{VgsxSNPeff^{2}}{Vp}x100$. In this algorithm (adapted from Xu 2013), the product $VgsxSNPeff^{2}$ is the Vg∗ associated with the variation in root-galling or egg-masses phenotypes in tested F${}_{2}$ and 2 F${}_{2:3}$ populations. To estimate the narrow-sense heritability ($h^{2}=\frac{Va}{Vp}$), the genetic variance (Vg∗=Va+Vd) was partitioned into additive and dominance variances, and the Va component was used to compute the $h^{2}$ of the trait. Root-galling data of seven F${}_{2}$ populations (populations in Table 1 plus their subsets) and parental genotypes were used to perform midparent-offspring regression analysis, and four mapping populations (two F${}_{2}$ and two F${}_{2:3}$, Exps. 1, 4, 8 and 9, Table 1) were used to derive genetic variances (Vg∗) within the QTL regions, influencing the response to galling and egg-mass production. Allelic relationships between the *Rk* locus present in cv. CB46 (Roberts *et al.* 1995; Hall and Frate1996; Roberts *et al.* 1996; Roberts *et al.* 1997; Ehlers *et al.* 2009; Huynh *et al.* 2016) and the genetic determinants of resistance in FN-2-9-04 were determined using the four F${}_{2}$ population sets of CB46 x FN-2-9-04 phenotyped with *M. incognita* isolate Project 77 and *M. javanica* infestation (Table 1).

### Genotyping and QTL mapping

Leaf samples were collected from parents and each of 119 and 137 F${}_{2}$ lines of populations CB46-Null x FN-2-9-04 and CB46 x FN-2-9-04, respectively (Exp. 1, 5, Table 1) 30 days after transplanting and dried in plastic ziploc bags containing silica gel packs. Genomic DNA was extracted from dried leaves using Plant DNeasy (Qiagen protocol) and quantified using Quant-iTTM dsDNA Assay Kit and fluorescence measured using a microplate reader. In addition, each F${}_{2}$ plant of population CB46-Null x FN-2-9-04 was selfed to generate F:23 seeds for field phenotyping (Table 1). The 119 F${}_{2}$ lines are part of the 163 lines tested for egg-mass production (Exp. 1) and transplanted for root-galling assay (Exp. 4, Table 1).

Each DNA sample was assayed for single nucleotide polymorphism (SNP) using the Cowpea iSelect Consortium Array containing 51128 SNPs (Muñoz-Amatriaín *et al.* 2017). The SNP data were filtered for quality as follows: (i) elimination of SNPs with > 20% missing data; (ii) elimination of monomorphic SNPs; (iii) elimination of SNPs with minor allele frequency (MAF) < 0.4 and < 0.3 for populations CB46-Null x FN-2-9-04 and CB46 x FN-2-9-04, respectively; iv) and elimination of duplicated lines. No loci were detected with non-parental alleles.

Linkage-maps of the CB46-Null x FN-2-9-04 and CB46 x FN-2-9-04 F${}_{2}$ populations were constructed with MSTmap (Wu *et al.* 2015), and linkage groups were determined at LOD threshold = 10 and marker placement followed the Kosambi mapping function. The options “no mapping size threshold” and “no mapping distance threshold” were fixed at 2 units and 10 cM, respectively. In addition, the no mapping distance threshold option was set at 15 cM and the detection of genotyping errors was not solicited. The linkage groups of the final genetic map were numbered and ordered following the cowpea consensus genetic map order (Muñoz-Amatriaín *et al.* 2017) and the cowpea pseudomolecules (Lonardi *et al.* 2017 in preparation; https://phytozome.jgi.doe.gov/). Also, the cowpea reference genome was used to determine the physical positions of the SNPs and the QTL associated with the traits (Lonardi *et al.* 2017 in preparation; https://phytozome.jgi.doe.gov/).

QTL mapping was performed using five phenotypic data sets comprising two F${}_{2}$ populations of crosses CB46-Null x FN-2-9-04 and CB46 x FN-2-9-04, and two F${}_{2:3}$ populations of cross CB46-Null x FN-2-9-04 (Exps. 1, 4, 5, 8 and 9, Table 1). QTL analysis was performed following the mixed-model for QTL mapping described by Xu (2013) using RStudio v1.1.442, and significant QTL were declared using Bonferroni adjusted threshold value -log (*P*-value) at *P* < 0.05. Reported QTL regions associated with resistance were based on the SNP markers with the most significant threshold values. In the mixed-model for QTL analysis (Xu 2013), the analysis comprises three input data sets; phenotypic and genotypic data sets, and a kinship data set matrix generated using genotypic data. The phenotypic response is associated with polygenic and marker effects; and these components are considered as random and fixed effects, respectively. The polygenic effect (total genetic variance) influencing the phenotype is further partitioned in to additive, dominance and epistatic effects. The proportion of phenotypic response explained comprises the genotypic and marker effects $Vp*=\frac{VgsxSNPeff^{2}}{Vp}x100$ (see details in the previous section). All phenotypic data sets comprised raw phenotypic data.

### Data Availability

All F${}_{2}$ and F${}_{2:3}$ populations and root-knot nematode isolates are available upon request. Phenotypic and genotypic data are included in data (D) files 1 - 9. These data files, including their description, and supplementary tables and figures are available at Figshare: https://doi.org/10.25387/g3.7324211.

## Results

### Linkage and QTL mapping

The linkage map of the F${}_{2}$ population CB46-Null x FN-2-9-04 (n = 119) contained 17208 polymorphic SNP markers distributed on 11 chromosomes and spanned 985.89 cM (Supplementary file S1A). Of the total SNPs, 90.79% (15624 SNPs) were mapped on the cowpea consensus genetic map (Muñoz-Amatriaín *et al.* 2017), while 9.21% (1585 SNPs) were unique to this population, and this portion corresponds to 2.5% of SNPs not mapped to the cowpea pseudomolecules. The linkage map comprised 1392 bins distributed at an average density of 1 bin per 0.71 cM. The linkage map of the F${}_{2}$ population CB46 x FN-2-9-04 (n = 137 lines) contained a total of 17903 polymorphic SNPs and spanned 1158.68 cM (Supplementary file S1B). Of these SNPs, 97.6% (17465 SNPs) mapped to the cowpea consensus genetic map, while 9.4% (1675 SNPs) are not part of the cowpea consensus genetic map, and this portion makes 2.4% of the total SNPs not mapped on the cowpea pseudomolecules (Lonardi *et al.* 2017 in preparation; https://phytozome.jgi.doe.gov/).

**Figure 1:**
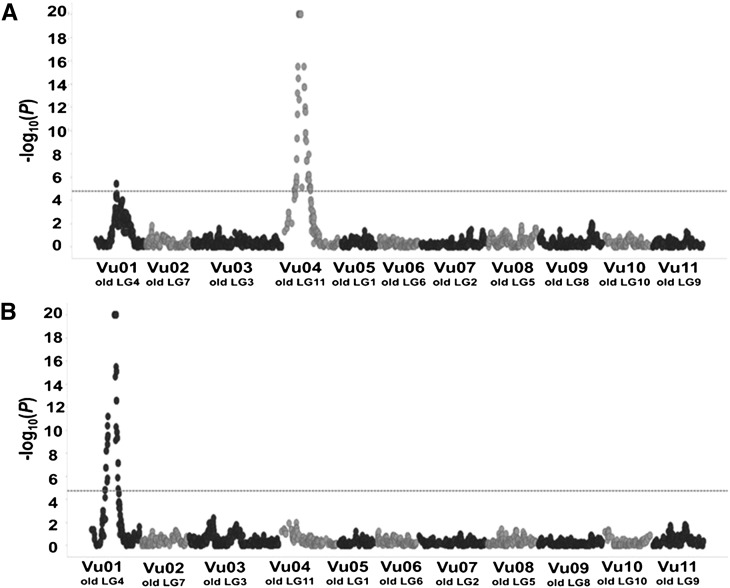
Genomic localization of QTL associated with resistance to root-galling (RG) by: A, avirulent *M. incognita* and B, aggressive *M. javanica*. The QTL were detected in the CB46-Null x FN-2-9-04 F${}_{2:3}$ population phenotyped for RG under field infestation. Horizontal dashed line represents the Bonferroni threshold of significance at *P* < 0.05 [-log(*p*) = 4.8]. Old LG represents former cowpea linkage group numbering and Vu indicates the new cowpea linkage group numbering based on the cowpea pseudomolecules (Lonardi *et al.* 2017).

**Figure 2:**
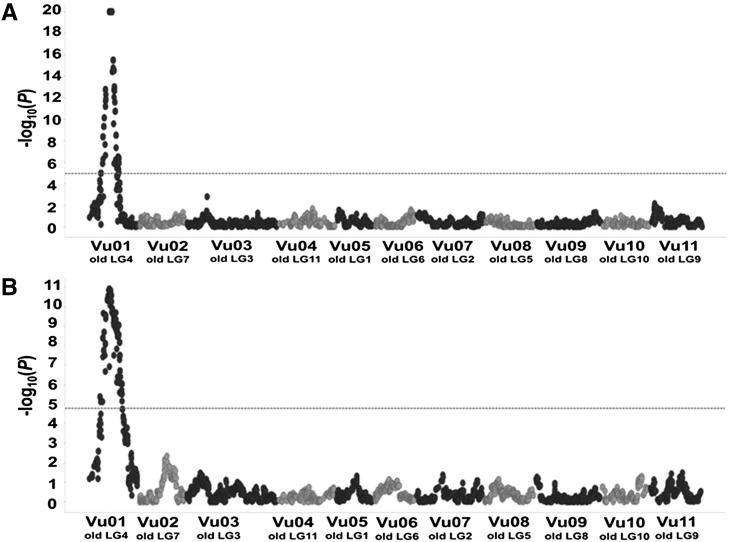
Genomic localization of QTL associated with resistance to A, root-galling (RG) and B, egg-mass production (EM) by aggressive *M. javanica*. The QTL were detected in the F${}_{2}$ population CB46-Null x FN-2-9-04 phenotyped for RG in the greenhouse and for EM in seedling growth-pouch inoculations, respectively. Horizontal dashed-line represents the Bonferroni threshold of significance at *P* > 0.05 [-log(*p*) (A and B = 4.9 and 4.8, respectively). Old LG represents former cowpea linkage group numbering and Vu indicates the new cowpea linkage group numbering based on the cowpea pseudomolecules (Lonardi *et al.* 2017).

QTL analysis revealed two major QTL associated with resistance to root-galling (RG) and egg-mass (EM) production in FN-2-9-04 (Table 2; Figures 1 and 2); these QTL were mapped on chromosomes Vu01 and Vu04 of the CB46-Null x FN-2-9-04 population and chromosome Vu01 of the CB46 x FN-2-9-04 population. The QTL region on Vu01 consistently mapped almost within the same genomic location using F${}_{2}$ and F${}_{2:3}$ populations phenotyped under greenhouse, seedling-growth pouch and field conditions using two RKN isolates (Table 2; Supplementary file S1C). Two QTL controlling resistance to RG by avirulent *M. incognita* Beltran were detected and mapped on Vu01 and Vu04 (*P* < 0.05, threshold value -log(*p*) = 4.8) (Figure 1A) of the CB46-Null x FN-2-9-04 F${}_{2:3}$ population. The resistance QTL on Vu01 mapped to position 34.4 cM which spanned 0.1 Mb (28855569 - 28960128 bp) on the cowpea pseudomolecules (Supplementary file S1C) between flanking markers 2..04038 and 2..04039; it accounted for 33% of the total phenotypic variation (*Vp*) of the RG resistance response and had a likelihood of occurrence expressed by -log10(*p*) = 5.4 (Table 2). Input data files used for this QTL discovery are D1 and D2.

The resistance QTL on Vu01 (Figure 1A) detected under plant infection by avirulent *M. incognita*, exhibited additive and dominance effects of -1.3 and -0.6, respectively, and the degree of dominance, measured as a ratio between dominance and additive effects (D/A), indicated that the resistance in this QTL has partial dominant effect (D/A = 0.5) (Table 2). A second resistance QTL associated with response to the avirulent *M. incognita* was detected on Vu04 (Figure 1A, Table 2) at chromosome position 24.7 - 27.6 cM of the CB46-Null x FN-2-9-04 F${}_{2:3}$ population and spanned 2.9 cM which corresponds to approximately 1 Mb (3141521 – 4138458 bp) on the cowpea pseudomolecules (Supplementary file S1C), and it was flanked by SNP markers 2..44685 and 2..10583 (Table 2). This QTL explained 73.3% of the total *Vp* of the resistance response, and it had an infinite likelihood of occurrence which was represented by -log10(*p*) = 20 (Table 2). In addition, the additive (A = -2) and dominance (D = -1) effects of the QTL on Vu04 were slightly higher than those of the QTL on Vu01, but both QTL showed the same degree of dominance (D/A = 0.5).

On Vu01, an additional genomic region controlling resistance to *M. javanica* RG (Figures 1B; 2A) and EM production (Figure 2B) was consistently mapped on the same chromosomal region of the CB46-Null x FN-2-9-04 F2 and F${}_{2:3}$ populations using RG and EM phenotypic data from field, greenhouse and seedling-growth pouch experiments (Table 2). The *M. javanica* root-galling resistance QTL mapped to positions 30.3 - 38.7 cM and 27.7 - 42.0 cM on Vu01 using F${}_{2}$ (greenhouse experiment - input data files D1 and D5) and F${}_{2:3}$ (Field experiment - input data files D1 and D3) populations from the CB46-Null x FN-2-9-04 cross, respectively. These genomic regions spanned 8.4 and 14.3 cM, which correspond to 4.4 (26617356 - 31070755 bp) and 6.2 Mb (25784028 - 31953708 bp) on the cowpea pseudomolecules (Supplementary file S1C) and were flanked by SNP markers 2..32677 - 2..19840 and 2..47796 - 1..0027, respectively (Table 2). In both F${}_{2}$ and F${}_{2:3}$ populations, the RG resistance QTL was detected with infinite likelihood represented by -log10(*p*) = 20 (Figures 1B, 2A, Table 2). The percent of total phenotypic variation in RG explained by the QTL effect in the F${}_{2:3}$ (PVE = 95.1%) was higher than in the F${}_{2}$ (PVE = 47.2%), while the contributions of the additive and dominance effects in the total phenotypic variation in the F${}_{2}$ and F${}_{2:3}$ were similar (Table 2). Also, the degree of dominance in both generations were comparable, D/A = 0.4 and 0.3, respectively, indicating resistance with partial dominance.

The QTL on Vu01 associated with resistance to *M. javanica* reproduction (EM - input data files D1 and D4) mapped to position 31.5-36.9 cM of the CB46-Null x FN-2-9-04 F${}_{2}$ population (Figure 2B; Table 2). This QTL spanned 5.5 cM which corresponds to 2.7Mb (27254299 - 29984745 bp) on the cowpea pseudomolecules (Supplementary file S1C), and it was flanked by SNP markers 2..21671 and 2..07103. This QTL accounted for 34.1% of the total phenotypic variation in EM production with additive and dominance effects of 17.1 and 7.8, respectively; the gene action measured within the same QTL region indicated resistance with partial dominance (D/A = 0.5). Although this QTL was detected with high likelihood, -log10(*p*) = 10.9 (critical threshold = 4.8) (Figure 2B), it was lower than that observed for the RG QTL (Table 2).

**Figure 3:**
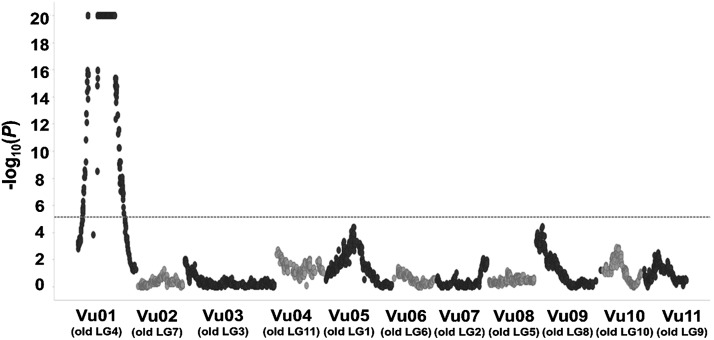
Genomic localization of QTL associated with resistance to root-galling induced by aggressive *M. javanica*. The QTL was detected in the CB46 x FN-2-9-04 F2 population phenotyped for RG in the greenhouse. Horizontal dashed-line represents the Bonferroni threshold of significance at *P* < 0.05 [-log(*p*) = 5.1. Old LG represents former cowpea linkage group numbering and Vu indicates the new cowpea linkage group numbering based on the cowpea pseudomolecules (Lonardi *et al.* 2017).

QTL mapping using the F${}_{2}$ population of CB46 x FN-2-9-04 (input data files D6 and D7) validated that the genomic region on Vu01 is associated with resistance to *M. javanica* RG (Figure 3; Table 2). This Vu01 genomic region was mapped to position 19.2-72.9 cM in the CB46 x FN-2-9-04 F2 population, and it spanned 53.7 cM which corresponds to 13.5 Mb (20889089 - 34401992 bp) on the cowpea pseudomolecules with flanking SNP markers 2..53036 - 2..18359 (Table 2; Supplementary file S1C). The QTL on Vu01 explained 65.9% of the total phenotypic variation in *M. javanica* root-galling, and the contribution of the additive and dominance effects were 2.7 and 2.1, respectively. The estimated gene action within this region indicated resistance with partial dominance (D/A = 0.8) (Table 2). This QTL was detected with high likelihood, -log10(*p*) = 20 (critical threshold = 5.1) (Figure 3). In addition, a genomic region associated with resistance to *M. javanica* EM production was mapped on Vu01 of the CB46 x FN-2-9-04 F${}_{2}$ (input data files D8 and D9) at position 46.7 – 53.5 cM, and it spanned 6.8 cM corresponding to 3.2 Mb (27254299 - 30434421 bp) on the cowpea pseudomolecules flanked by SNP markers 2..21671 – 2..12209. This QTL explained 24.7% of the total phenotypic variation in *M. javanica* EM production. (Table 2; Supplementary file S1C).

### Inheritance of resistance in FN-2-9-04

**Figure 4:**
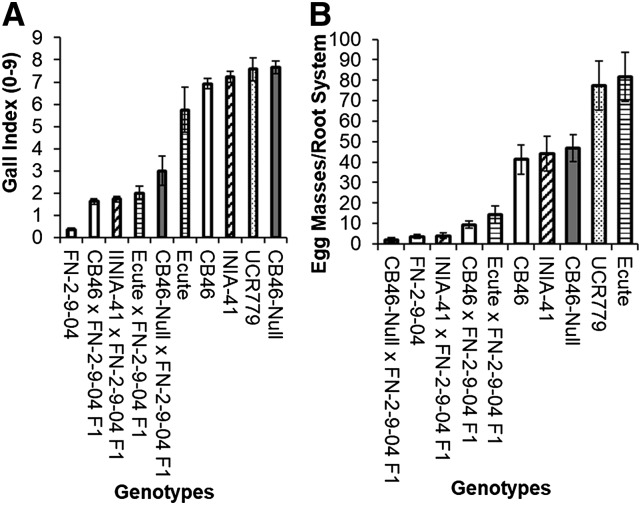
Mean response of F${}_{1}$ populations and their parents to: A, root-galling and B, egg-mass production by *M. javanica* in greenhouse-pot and seedling growth-pouch inoculations, respectively. Bars represent +/− SE.

Figures 4A and 4B show the response of four F${}_{1}$ populations and their parental genotypes to root-galling (RG) and egg-mass (EM) production, respectively by *M. javanica*. All recurrent parents (Ecute, CB46, INIA-41 and CB46-Null) exhibited susceptible phenotypes for RG and EM, and their mean RG scores and EM scores ranged from 5.8 to 7.7 and 41 to 82, respectively, whereas the resistant parent, FN-2-9-04 had mean RG and EM scores of 0.4 and 4, respectively.

All F${}_{1}$ populations were resistant to *M. javanica* (Figure 4), with mean RG and EM scores below the mid-parent RG and EM score (GI = 6.9 and EM = 53). The CB46-Null x FN-2-9-04 F${}_{1}$ had the highest mean RG (GI = 3) of the four F${}_{1}$ populations. The observed differences in RG and EM between the resistant and susceptible parents were significant (*P* < 0.05), but the RG phenotype of the resistant parent was only different from F${}_{1}$ populations CB46-Null x FN-2-9-04 and Ecute x FN-2-9-04. The EM phenotypes of the resistant parent and F${}_{1}$ were not different. Significant differences among the genotypes were detected at GI = 1.3 and EM = 31.4 (Figure 4A and 4B).

**Figure 5:**
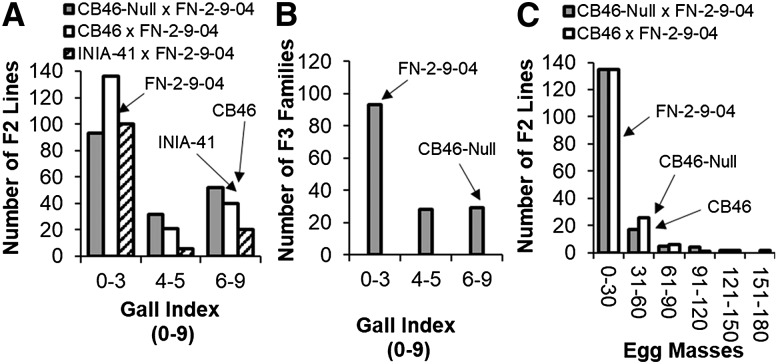
Distribution of root-galling responses in A, F${}_{2}$ populations (greenhouse), B, F${}_{2:3}$ population CB46-Null x FN-2-9-04 (field), and C, egg-mass production in F${}_{2}$ populations CB46-Null x FN-2-9-04 and CB46 x FN-2-9-04 (seedling growth-pouch) under *M. javanica* infestation.

The segregation of F${}_{2}$ (Figure 5A) and F${}_{2:3}$ (Figure 5B) populations for *M. javanica* RG response appeared to follow a bimodal distribution, skewed toward lower RG phenotype. Also, a bimodal segregation pattern was observed for *M. javanica* EM production in the CB46-Null x FN-2-9-04 and CB46 x FN-2-9-04 F${}_{2}$ populations (Figure 5C). In these same experiments, the average RG observed for parents CB46-Null, CB46, INIA-41 and FN-2-9-09 in greenhouse pots was 7.7, 6.9, 7.2 and 0.4, respectively. In the field experiment (Figure 5B), RG of 6.7 and 0.1 were observed for parents CB46-Null and FN-2-9-09, respectively, while egg-mass counts per root system equal to 46.7, 45 and 1.8 were observed for parents CB46-Null, CB46 and FN-2-9-09, respectively (seedling-growth pouches).

**Figure 6:**
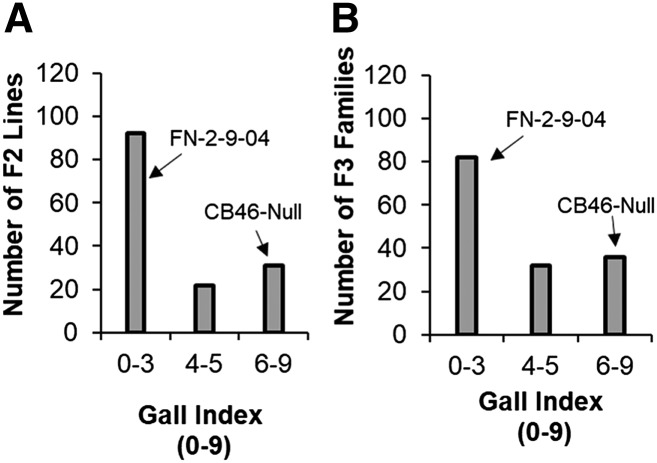
Distribution of root-galling response in the F${}_{2}$ (A) and F${}_{2:3}$ (B) populations of CB46-Null x FN-2-9-04 under field infestation with avirulent *M. incognita* isolate Beltran.

A similar pattern of root-galling distribution was observed in F${}_{2}$ (Figure 6A) and F${}_{2:3}$ (Figure 6B) populations of CB46-Null x FN-2-9-04 under field infestation by avirulent *M. incognita* Beltran. This segregation pattern was consistent across all phenotyping environments (greenhouse, field and seedling growth-pouches) and traits (RG and EM). Egg-mass phenotypes ranged from 0 – 180 (Figure 5C), and RG across environments and generations ranged from 0 – 9 (Figures 5 and 6). The resistant parent FN-2-9-04 had consistently lower (*P* < 0.05) RG compared to all susceptible parents. The average *M. incognita* root-galling indices for parents CB46-Null and FN-2-9-04 in the field experiment were 6.4 and 0, respectively.

**Figure 7:**
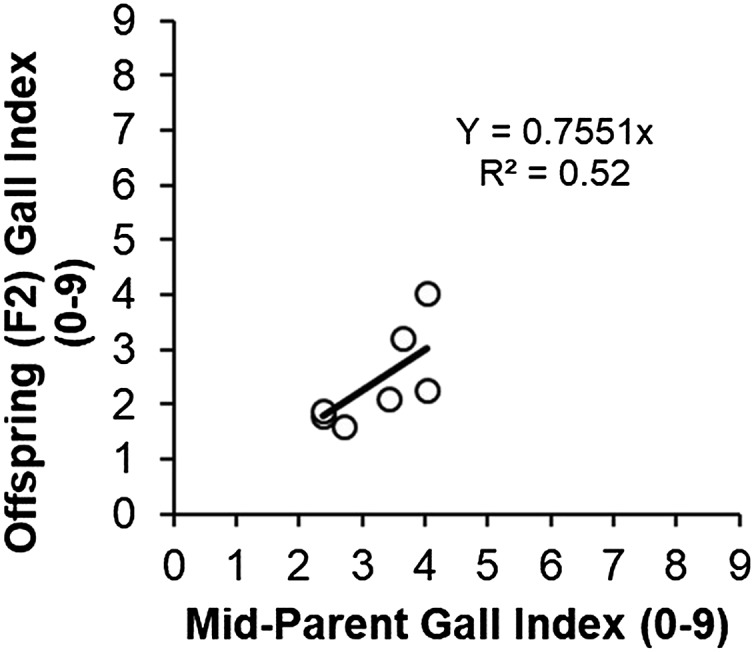
Midparent – offspring regression for F${}_{2}$ population means regressed on the midparent root-galling values.

The broad-sense heritability ($H^{2}$) of resistance to *M. javanica* root-galling estimated through regression of 7 field phenotyped F${}_{2}$ populations to the mean performance of their parents (CB46-Null, CB46, FN-2-9-04 and INIA-41,) was high (*b* = 0.76 ± 0.07, *P* = 0.00004) (Figure 7), while estimates of $H^{2}$ for the same trait computed using the genetic variance (Vg*) directly derived from the QTL region located on Vu01 were moderate (0.47) and high (0.95) for greenhouse and field phenotyped F${}_{2}$ and F${}_{2:3}$ populations, respectively. For these populations, the estimates of narrow-sense heritability ($h^{2}$) of RG were 0.33 and 0.71, respectively. Egg mass production (EM) response in the F${}_{2}$ had low $H^{2}$ (0.34) (Table 2) and $h^{2}$ (0.23). The estimated $H^{2}$ and $h^{2}$ for resistance to avirulent *M. incognita* RG were 0.33 and 0.23 on Vu01 and 0.73 and 0.49 on Vu04, respectively.

**Figure 8:**
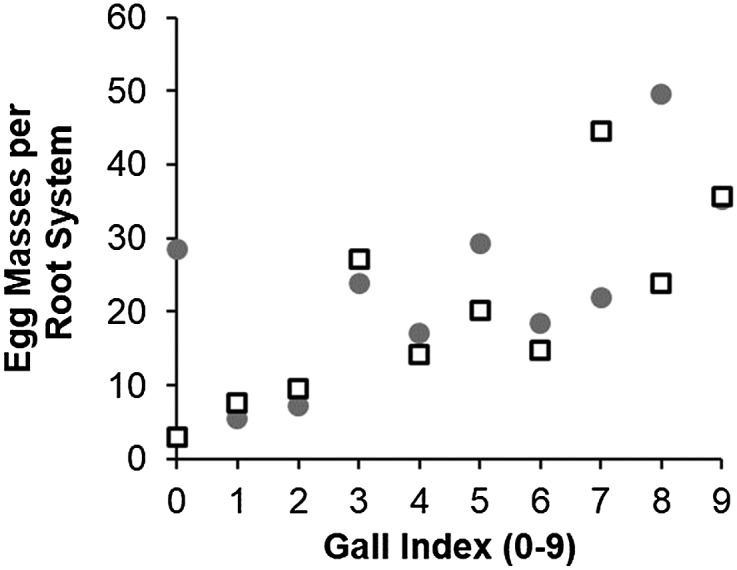
Correlation between *M. javanica* root-galling (greenhouse) and egg-mass production (seedling growth-pouch) in F${}_{2}$ populations Circle marker = CB46-Null x FN-2-9-04 (r = 0.62) and square marker = CB46 x FN-2-9-04 (r = 0.78).

Because the *M. javanica* RG and EM resistance QTL were co-located (Figures 1B, 2A and 2B), analysis of correlation between RG and EM responses was performed using RG and EM data of F${}_{2}$ populations CB46 x FN-2-9-04 and CB46-Null x FN-2-9-04. These traits were highly correlated in both populations, CB46 x FN-2-9-04 and CB46-Null x FN-2-9-04 (*r* = 0.78, *P* = 0.008 and *r* = 0.62, *P* = 0.06, respectively), although the correlation in the F${}_{2}$ population CB46-Null x FN-2-9-04 was not significant (*P* = 0.06) (Figure 8). The relationship between RG and EM in populations CB46 x FN-2-9-04 and CB46-Null x FN-2-9-04 was explained at 60.3% and 38.1%, respectively, based on the estimated coefficient of determination.

The 119 and 137 F${}_{2}$ lines of populations CB46-Null x FN-2-9-04 and CB46 x FN-2-9-04, respectively, assayed for 51128 SNP markers segregated for resistance-susceptibility to RG and EM within each mapped QTL, and it fit closely a ratio of 13:3 for phenotypic traits (Table 3). Also, a 3:1 ratio was significant, suggesting that the resistance at both QTL regions is mainly governed by one dominant gene or a combination of genes acting under dominant-recessive interaction. The fit to a 13:3 ratio could also indicate genetic distortion for a single dominant gene.

To validate the genetic models of segregation for resistance-susceptibility to avirulent *M. incognita* and *M. javanica*, gene enumerations were estimated at the mapped QTL regions associated with resistance to RG (Vu01 and Vu04) and EM production (Vu01) following the Castle (1921) algorithm. The estimates indicated that the resistance to avirulent *M. incognita* RG is under control primarily by 2 and 5 genes residing in QTL regions mapped on Vu04 and Vu01, respectively; whereas, the responses to *M. javanica* RG and EM production mapped on Vu01 are governed mainly by 2 genes each (Supplementary file S3).

**Figure 9:**
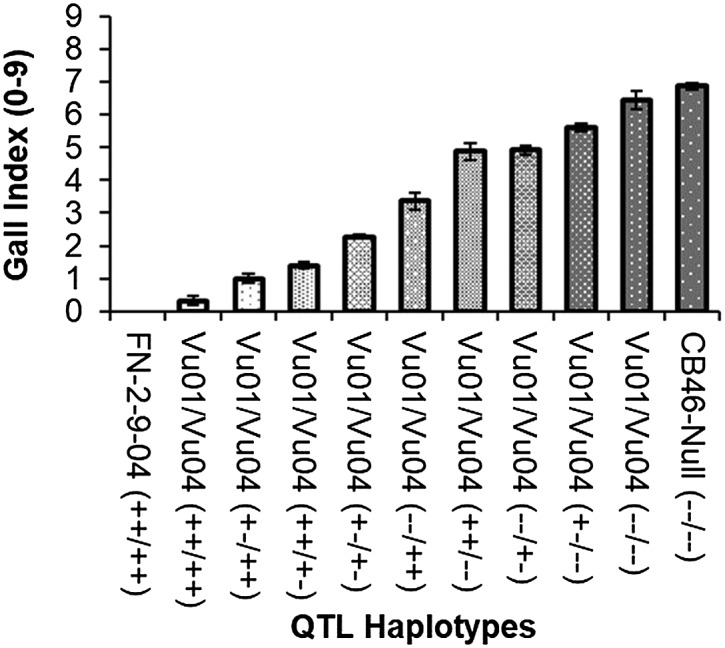
Avirulent *M. incognita* root-galling values for QTL allele combinations for the resistance traits in accession FN-2-9-04 mapped to Vu01 and Vu04 of the cowpea consensus genetic map. The zygosity status within each QTL is indicated by ++, +- and --, representing homozygous favorable, heterozygous and homozygous un-favorable, respectively, in each QTL. Bars are standard errors.

Because two QTL, on Vu01 and Vu04, were associated with resistance to avirulent *M. incognita* RG, analysis of QTL allele combinations were performed to understand the interaction of both QTL. Through SNP marker-trait association, the genotype (AA, AB and BB) of each of the 119 F${}_{2}$ lines was determined at the QTL regions on Vu01 and Vu04 associated with resistance to avirulent *M. incognita* RG, and each genotype was associated with the average RG phenotypic response of the corresponding F${}_{2:3}$. Based on this association, nine QTL combinations (Vu01/Vu04) (Figure 9) were derived by combining all possible haplotypes on Vu01 and Vu04 contributed from resistant (FN-2-9-04 – favorable allele donor) and susceptible (CB46-Null – non-favorable allele donor) parents.

Analysis of variance showed significant effect (*P* < 0.05) of combining QTL on avirulent *M. incognita* RG response; significant mean differences in RG phenotypes between genotypes carrying combined QTL were detected at gall index (GI) = 0.88. The resistant parent FN-2-9-04 [Vu01/Vu04(++/++)] did not show any root-galling (Figure 9), and its response was different (*P* < 0.05) from all genotypes carrying QTL haplotypes with favorable allele dosage different from this parent. Any of the genotypes carrying at least a single favorable allele on at least one of the chromosome regions had less galling than the susceptible parent CB46-Null [Vu01/Vu04(–/–)]. Absence of a single favorable allele in either chromosome predisposed the plants to root-galling, and substantial allele effect was observed for Vu04 [Vu01/Vu04(++/+-)] (Figure 9). At both loci the favorable alleles must be in the homozygous condition for fully effective *M. incognita* RG resistance.

### Resistance relationship Between CB46 and FN-2-9-04

**Figure 10:**
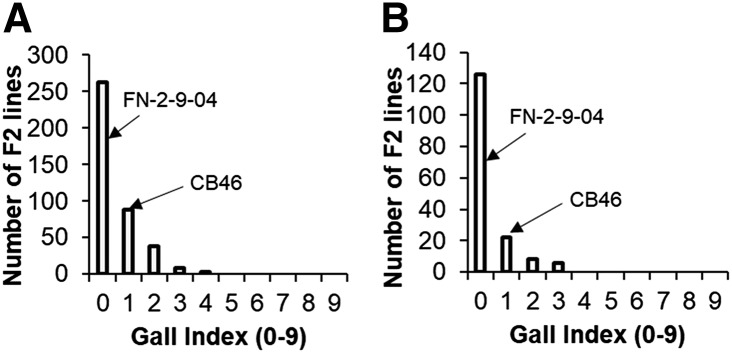
Distribution of root-galling response in the F${}_{2}$ population CB46 x FN-2-9-04 under field infestation by avirulent *M. incognita *(A): Coachella Valley Agricultural Research Station and (B): Kearney Agricultural Research and Extension Center, respectively.

The relationship between the root-galling and nematode reproduction resistance in accession FN-2-9-04 and resistance conferred by the *Rk* gene in CB46 (Huynh *et al.* 2016) was determined through allelism tests using F${}_{2}$ populations of CB46 x FN-2-9-04. In addition, analysis of similarity was performed between FN-2-09-04, CB46 and breeding line CB46-Null within the mapped QTL regions to identify putative haplotypes associated with resistance in FN-2-9-04. In 2015 (Table 1), 400 and 162 F${}_{2}$ plants plus parents were phenotyped for avirulent *M. incognita* root-galling under field infestation at CVARS and KARE, respectively. At both sites (Figure 10), all F${}_{2}$ plants were resistant with no obvious segregation for root-galling response between plants, indicating that FN-2-9-04 carries a resistance locus allelic to or equivalent to the *Rk* gene found in CB46. The average root-gall indices for CB46 and FN-2-9-04 were 0.7 and 0.2, respectively.

To validate the allelic relationship between resistance determinants conferring resistance to RKN in CB46 and FN-2-9-04, F${}_{2}$ population subsets of CB46 x FN-2-9-04 were also phenotyped for resistance to *M. javanica* RG and EM, since these parents exhibited significant differences in *M. javanica* RG and EM production responses (Figure 4). Using 197 and 172 F${}_{2}$ lines for RG and EM phenotyping, respectively (Table 1), segregation occurred for *M. javanica* RG and EM in these F${}_{2}$ populations as shown in Figures 5A and 5C. Analysis of similarity between FN-2-09-04, CB46 and CB46-Null within the Vu04 genomic region associated with avirulent *M. incognita* RG resistance (Table 2; Figure 1A) revealed a putative haplotype associated with the resistance (Supplementary file S4). The location of the *Rk* locus on Vu04 identified in CB46 (Huynh *et al.* 2016) overlapped with the resistance region on the same chromosome in FN-2-9-04 within 2.9 cM of the CB46-Null x FN-2-9-04 F2 population and within 1.59 cM on the cowpea consensus genetic map (Muñoz-Amatriaín *et al.* 2017), corresponding to approximately 1 Mb on the cowpea pseudomolecules. Within this region, based on SNP marker haplotypes, FN-2-9-04 is 39% identical to CB46 and completely different from CB46-Null (identity = 0%) which is 60% identical to CB46. Conversely, in the region on Vu01 where an additional resistance QTL was detected in FN-2-09-04 (Table 2; Figures 1B, 2A, 2B), this resistant parent shares no SNP haplotype similarity with either CB46 or CB46-Null (identity = 0%), whereas CB46 and CB46-Null are 100% identical.

## Discussion

Characterization of the resistance to avirulent *M. incognita* and aggressive *M. javanica* present in cowpea accession FN-2-9-04 from Mozambique revealed that the resistance is determined by two major QTL which were mapped on chromosomes Vu01 (old LG4) and Vu04 (old LG11) in the CB46-Null x FN-2-9-04 populations and on Vu01 in the CB46 x FN-2-9-04 population.

The QTL mapped on Vu04 overlaps with the previously mapped genomic region which harbors the *Rk* resistance locus (Huynh *et al.* 2016), suggesting that the *Rk* locus is also present in FN-2-9-04. In our previous RKN resistance QTL mapping of *QRk-vu4.1* (old *QRk-vu11.1*) (*Huynh et al.* 2016), this region associated with the *Rk* resistance spanned about 8.35 cM compared to 2.9 cM in this study. This difference in mapping resolution is attributed in part to the current availability of the high-density SNP genotyping platform and high-density cowpea consensus genetic map (Muñoz-Amatriaín *et al.* 2017). If the genomic region harboring the *Rk* locus is a multi-allelic or multi-gene locus, the overlap between *QRk-ʋu4.1* and the QTL mapped in this study on Vu04 indicates that the resistance alleles are within 2.9 cM interval of the CB46-Null x FN-2-9-04 population corresponding to approximately 1 Mb on the cowpea pseudomolecules. This locus provides effective resistance against avirulent *M. incognita* populations. The resistance to avirulent *M. incognita* present on Vu01 in FN-2-9-04 is confined to 0.1 Mb of the cowpea pseudomolecules, and its relatively low contribution to the total phenotypic variation in root-galling response (33%) compared to the resistance in Vu04 (73.3%) supports that the resistance in Vu04 is the main resistance for this nematode although both are required in the FN-2-9-04 background for fully effective resistance. The estimated values of contribution of each resistance QTL to the total phenotypic variance (Vu01 + Vu04; 33% + 73.3%) give a reliable indication of activity of each resistance QTL to the observed root-galling phenotypic response, with the excess in estimation attributed to error.

The resistance to *M. javanica* in FN-2-9-04 consistently mapped to Vu01 using root-galling and egg-mass production phenotypic data from F${}_{2}$ and F${}_{2:3}$ populations phenotyped under distinct environmental conditions (greenhouse, growth chamber and field). The QTL associated with resistance to *M. javanica* egg-mass production was co-located with the QTL controlling root-galling response, and based on the physical positions, on the cowpea pseudomolecules, of the mapped resistance QTL, the resistance to *M. javanica* root-galling and egg-mass production are confined within 6.2 Mb. The resistance QTL on Vu01 is distinct from the *Rk* locus (*QRk-ʋu4.1*, Huynh *et al.* 2016) which was mapped on Vu04, also it is distinct from the recently mapped RKN resistance locus on Vu11 (Old LG9) which also confers resistance to *M. javanica* (Santos *et al.* 2018). Therefore, it represents a novel RKN resistance QTL in cowpea designated here as *QRk-vu1.1*.

The response of four F${}_{1}$ populations to root-galling and egg-mass production relative to the resistant parent, and the skewed segregation of these nematode-induced phenotypes in the F_2_ and F${}_{2:3}$ populations indicated that these responses are under control by major genes with partial dominance effects, as also indicated by the estimated degrees of dominance (D/A). Resistance to RKN under control by major genes with partial dominance effect has been reported in several studies (Ali *et al.* 2014; Huynh *et al.* 2016).

Analysis of segregation for resistance against *M. javanica* and avirulent *M. incognita* through marker-trait association better fit a 13:3 ratio expected for a genetic control under a single dominant gene plus a recessive gene on both Vu01 and Vu04, also suggesting that the major genes controlling resistance are putatively aided by minor/recessive genes, and collectively in a dominant-recessive interaction to confer substantially stronger, broad-based resistance than that conferred by the *Rk* gene alone. A similar genetic phenomenon of major gene and minor/recessive gene interaction was described in cowpea cultivar CB27, where gene *Rk* acts together with a recessive gene to enhance and broaden root-knot nematode resistance (Ehlers *et al.* 2000a). The data also fit a 3:1 ratio expected for a single major gene, and the better fit to the 13:3 of the SNP haplotypes could represent genetic distortion within each locus. However, using the Castle (1921) algorithm for gene enumeration, the estimates also supported that two genes on Vu01 and two genes on Vu04, may be responsible for the resistance against *M. javanica* and avirulent *M. incognita*, respectively, but the estimates of genes involved in resistance against avirulent *M. incognita* on Vu01 did not support the observed segregation for resistance. The extent of genetic distortion in these regions or multi-allelic effects require further study.

Estimates of heritability of resistance in FN-2-9-04 to avirulent *M. incognita* and aggressive *M. javanica* in the F${}_{2}$ generation using greenhouse phenotypic data were lower than those estimated in the F${}_{2:3}$ generation using phenotypic data from field experiments. This can be accounted for by the segregation in both populations and because greenhouse phenotyping is less variable compared to field testing. The estimates of narrow-sense heritability of resistance to root-galling induced by both RKN species were in the range 0.23 – 0.71, indicating that the resistance in FN-2-9-04 can be transferred successfully into elite cowpea cultivars to broaden the genetic base of root-knot resistance which currently relies on the *Rk* gene. The resistance response to *M. javanica* reproduction had lower heritability estimates (*H${}^{2}$* = 0.25 and 0.34; *h${}^{2}$* = 0.17 and 0.24) compared to those for *M. javanica* induced root-galling (*H${}^{2}$* = 0.47 - 0.95; *h${}^{2}$* = 0.33 - 0.71), which could be due to egg-mass production data being generally more variable compared to root-galling data. High correlation between root-galling and nematode reproduction responses, and the co-location of resistance QTL associated with both phenotypes suggests that both traits may be governed by the same genes determining resistance. Similarly, significant correlation between root-galling and reproduction phenotypes in cowpea recombinant inbred populations was reported by Huynh *et al.* (2016) for the *Rk* locus on Vu04. In contrast, in lima bean (*Phaseolus lunatus* L.) the responses to root-galling and nematode reproduction were reported to be under control by independent genetic factors (Roberts *et al.* 2008). Since genetic factors explained 38.1 and 60.3% of the association between root-galling and egg-mass production in this study, these data suggest that although the genomic regions governing both traits are co-located, these traits may be under distinct regulatory mechanisms, or that the resistance to both traits may reside within a multi-allelic locus or tandemly arranged loci.

The heritability of resistance to avirulent *M. incognita* root-galling comprised two components, one on Vu01 (*H${}^{2}$* = 0.33; *h${}^{2}$* = 0.23,) and the other on Vu04 (*H${}^{2}$* = 0.73; *h${}^{2}$* = 0.49) indicating that the major locus for this resistance in FN-2-9-04 is housed on Vu04, and it is aided by the additional locus on Vu01 with low resistance heritability. Also, the differential activity between the resistance loci on Vu01 and Vu04 points to specificity of resistance to avirulent *M. incognita* and *M. javanica*. Huynh *et al.* (2016) reported that, although the QTL harboring the *Rk* locus had a significant effect on controlling both avirulent *M. incognita* and *M. javanica*, its resistance activity was lower against *M. javanica*. Marker-trait association analysis in the current study indicated that resistances on both Vu01 and Vu04 are required for effective resistance under avirulent *M. incognita* infestation.

The allelism test between CB46 and FN-2-9-04 revealed a lack of resistance segregation in the CB46 x FN-2-9-04 F${}_{2}$ population under avirulent *M. incognita* infestation, indicating that both parents carry the same major gene *Rk* locus previously mapped by Huynh *et al.* (2016) on Vu04 (old LG11) of the cowpea consensus genetic map (Muñoz-Amatriaín *et al.* 2017), also supporting that the resistance mapped in this study on Vu04 corresponds to the *Rk* locus. *Rk* was the first identified RKN resistance locus in cowpea, and it has been bred into many commercial cowpea cultivars (Fery and Dukes 1980; Helms *et al.* 1991; Ehlers *et al.* 2009). In contrast, the segregation found in F${}_{2}$ population CB46 x FN-2-9-04 for *M. javanica* root-galling and reproduction responses, and the mapping of resistance QTL for root-galling and egg-mass production confirmed that the heightened and broad-based resistance response in FN-2-9-04 relative to CB46 is conferred by novel resistance determinants located on Vu01.

Flanking markers associated with the mapped genomic regions on Vu01 and Vu04 can be used to assist the introgression of the resistance into elite cowpea cultivars. In particular, the novel resistance detected on Vu01 confers the most effective *M. javanica* resistance known to date in cowpea. The resistance on Vu01 appears to be more specifically effective against aggressive *M. javanica*, while both the Vu01 and Vu04 QTL have activity against avirulent *M. incognita*, but with the QTL on Vu04 playing the major role in resistance. This was also demonstrated by QTL pyramiding of resistance on Vu01 and Vu04. Thus, both resistance QTL on Vu01 and Vu04 are responsible for the strong and broad-based resistance observed in FN-2-9-04, which is more effective than the narrow-based resistance provided by the *Rk* gene alone. The mechanism of resistance displayed by this novel broad-based resistance is yet to be determined.

The genetic linkage maps of the F${}_{2}$ populations CB46-Null x FN-2-9-04 and CB46 x FN-2-9-04 are additional valuable genetic resources, especially because they are the first cowpea linkage maps constructed using a genotype from the cowpea gene-pool II from southeastern Africa (Huynh *et al.* 2013), and because 9.2% of the 17209 SNP markers on the CB46-Null x FN-2-9-04 map were unique to this population and were not mapped on the most recent version of the cowpea consensus genetic map (Muñoz-Amatriaín *et al.* 2017).
